# Long-Term Reciprocal Gene Flow in Wild and Domestic Geese Reveals Complex Domestication History

**DOI:** 10.1534/g3.120.400886

**Published:** 2020-07-01

**Authors:** Marja E. Heikkinen, Minna Ruokonen, Thomas A. White, Michelle M. Alexander, İslam Gündüz, Keith M. Dobney, Jouni Aspi, Jeremy B. Searle, Tanja Pyhäjärvi

**Affiliations:** * Department of Ecology and Genetics, PO Box 3000, Fi-90014 University of Oulu, Finland; † Department of Ecology and Evolutionary Biology, Cornell University, Ithaca, New York 14853; ‡ CMPG, University of Bern, Baltzerstrasse 6, 3012 Bern, Switzerland; § University of York, BioArCh, Environment Building, Wentworth Way, Heslington, York, YO10 5NG, UK; ** Department of Biology, Faculty of Arts and Sciences, University of Ondokuz Mayis, Samsun, Turkey; †† Department of Archaeology, Classics and Egyptology, University of Liverpool, 12–14 Abercromby Square, Liverpool L69 7WZ, UK; ‡‡ Department of Archaeology, University of Aberdeen, St Mary’s, Elphinstone Road, Aberdeen, AB24 3UF, UK; §§ Department of Archaeology, Simon Fraser University, Burnaby, B.C. V5A 1S6, 778-782-419, Canada

**Keywords:** *Anser anser*, domestication, domestic goose, hybridization, population genomics

## Abstract

Hybridization has frequently been observed between wild and domestic species and can substantially impact genetic diversity of both counterparts. Geese show some of the highest levels of interspecific hybridization across all bird orders, and two of the goose species in the genus *Anser* have been domesticated providing an excellent opportunity for a joint study of domestication and hybridization. Until now, knowledge of the details of the goose domestication process has come from archaeological findings and historical writings supplemented with a few studies based on mitochondrial DNA. Here, we used genome-wide markers to make the first genome-based inference of the timing of European goose domestication. We also analyzed the impact of hybridization on the genome-wide genetic variation in current populations of the European domestic goose and its wild progenitor: the graylag goose (*Anser anser*). Our dataset consisted of 58 wild graylags sampled around Eurasia and 75 domestic geese representing 14 breeds genotyped for 33,527 single nucleotide polymorphisms. Demographic reconstruction and clustering analysis suggested that divergence between wild and domestic geese around 5,300 generations ago was followed by long-term genetic exchange, and that graylag populations have 3.2–58.0% admixture proportions with domestic geese, with distinct geographic patterns. Surprisingly, many modern European breeds share considerable (> 10%) ancestry with the Chinese domestic geese that is derived from the swan goose *Anser cygnoid*. We show that the domestication process can progress despite continued and pervasive gene flow from the wild form.

Reproductive isolation is a defining feature of speciation and yet hybridization between species is an important general phenomenon in evolution (Arnold 2004; Abbott *et al.* 2013). Among birds, the Anseriformes (ducks, geese, and swans) show particularly pervasive hybridization, 41.6% to > 60% of species hybridizing with each other (Grant and Grant 1992; Ottenburghs *et al.* 2016a). Domestication generates differentiated gene pools and reproductive isolation between domestics and their wild progenitor, but hybridization between domestic and wild forms has been well demonstrated in both plants (Arnold 2004; Janzen *et al.* 2019) and animals (Godinho *et al.* 2011; Frantz *et al.* 2015). The impacts include genetic and trait enrichment of domestics, for instance, in chicken the acquisition of a yellow skin phenotype is a result of past mating between red junglefowl and gray junglefowl (Eriksson *et al.* 2008). In geese, a high tendency for hybridization between wild and domestic forms has also been suggested (Kuijken and Devos 1996; Heikkinen *et al.* 2015), creating an exciting opportunity to study the complex dynamics of hybridization and domestication.

The domestic geese of the world (European and Chinese forms) are derived from two different wild species: the graylag (*Anser anser*) and the swan goose (*Anser cygnoid*), respectively (Delacour and Scott 1959; Shi *et al.* 2006). *A. anser* and *A. cygnoid* shared a common ancestor about 3.4 Mya (Ottenburghs *et al.* 2016b) but are still able to hybridize (Ottenburghs *et al.* 2016a), and some domestic breeds are reportedly hybrid (Buckland and Guy 2002). The graylag has been divided into the western, nominate subspecies *A. a. anser* with a European breeding range and the eastern subspecies *A. a. rubrirostris* breeding further east, although the subspecific boundary is not well defined, and mitochondrial DNA has not been found to distinguish them (Heikkinen *et al.* 2015). Of these subspecies, *rubrirostris* is larger and lighter colored than *anser* (Cramp and Simmons 1977) and has a pink bill and cold pink legs in contrast to the orange bill and flesh-colored legs of *anser*, the bill color used as primary evidence in favor of the original domestication of *rubrirostris* (Kear 1990). As with all domesticates, domestic geese varieties are morphologically more diverse than their wild counterparts, particularly in plumage and body size (Buckland and Guy 2002).

The current knowledge about goose domestication relies largely on ancient texts and archaeological evidence. Questions about where and when domestication took place, the genetic changes associated with it and the later history of domestic geese, however, remain largely unresolved (Heikkinen *et al.* 2015). There are depictions from the New Kingdom of Egypt that suggest geese were already fully domesticated by the 18^th^ Dynasty (1450-1341 BCE). The earliest reliable reference to domestic geese in western Eurasia is Homer’s Odyssey (first half of 8^th^ century BCE) and geese were certainly well-established poultry by Roman times (Albarella 2005).

Genetic diversity in the mitochondrial DNA (mtDNA) of graylag and European domestic geese showed reduced diversity in the domestics (Heikkinen *et al.* 2015) which may result from an early domestication bottleneck or, alternatively, later breed formation. There is a particular mitochondrial haplogroup common in the domestics (Heikkinen *et al.* 2015), and archaeological domestic goose bones from the High Medieval (11^th^-13^th^ century CE) of Russia belonged to that haplogroup (Honka *et al.* 2018).

MtDNA relationships between extant Chinese and European domestic goose breeds confirm that the former, excluding one breed, have swan goose ancestry, whereas European domestic goose and the Chinese Yili breed have graylag ancestry (Shi *et al.* 2006; Sun *et al.* 2013; Ren *et al.* 2016). However, Chinese mtDNA haplotypes may occasionally occur in European domestics, and vice versa (Sun *et al.* 2013; Heikkinen *et al.* 2015).

Genomic data can be much more powerful than mtDNA in terms of inference about hybridization. For instance, New World cattle, along with their taurine ancestry have been shown genomically to have a greater proportion of indicine ancestry than previously assumed (McTavish *et al.* 2013) and genomic studies of domestic pigs have shown them to have received genetic input from wild boars (Frantz *et al.* 2015). Genomic studies of modern dog breeds also show an ancestry that can only be explained by gene flow from multiple regional wolf populations (Skoglund *et al.* 2015). Plant varieties are often shown to be the product of hybridization by genomic studies, for example maize (Hufford *et al.* 2013). Interpretation of genomic data are still challenging and for the study of domestic species and their interactions with their wild progenitors, it is best to apply genomics to infer jointly the genetic impact of initial domestication and subsequent hybridization of wild and domestic populations, as the latter can obscure domestic-wild genetic relationships and may also give a false impression of the location and number of times a species has been domesticated (van Heerwaarden *et al.* 2011; Marshall *et al.* 2014; Larson and Fuller 2014).

Here we investigate goose domestication history using genome-wide single nucleotide polymorphism (SNP) data from thousands of loci, obtained by genotyping-by-sequencing (GBS). We used 56 and 50 samples of graylag and domestic geese from a previous mtDNA study (Heikkinen *et al.* 2015), together with 2 new Turkish graylag and 25 new domestic specimens. We studied the interplay between domestication and hybridization by addressing the following questions: i) what is the extent of genetic differentiation among wild and domestic geese? ii) what is the approximate time of domestication? and iii) what is the role of intra- and interspecific hybridization in goose domestication history and iv) how does hybridization affect the genetic composition of modern populations?

## Materials and methods

### Sampling

**Figure 1:**
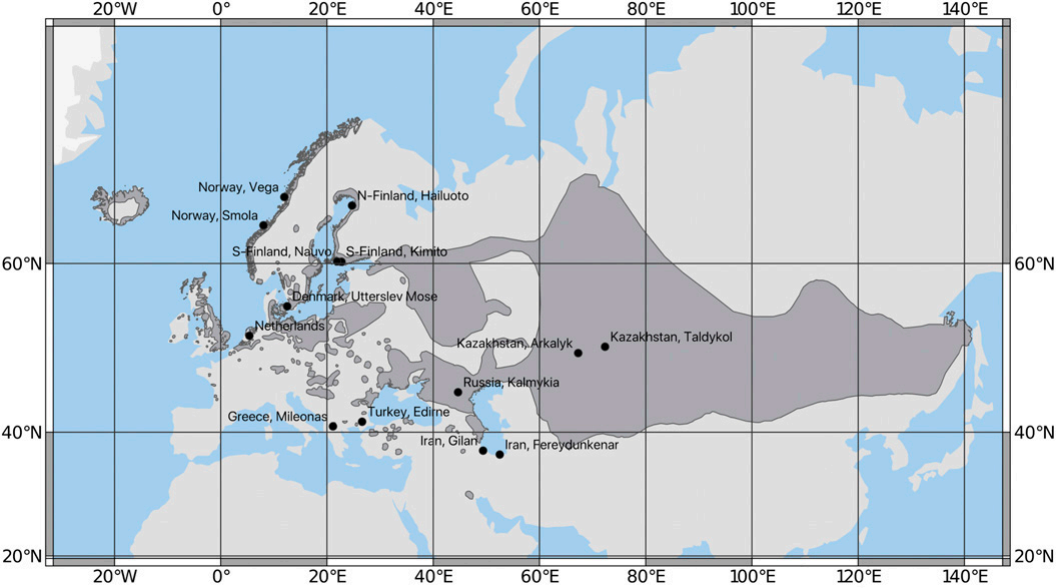
Map showing the sampling sites for wild graylags used in this study. The breeding area of the species is shown on darker gray. The sampling sites in Kazakhstan were combined for analyses (one sample per location) and the sampling sites in Southern Finland included combined samples from the geographically close sites of Västanfjärd, Nauvo (shown) and Kimito (shown). The Iranian samples were collected during the wintering season. Map modified from IUCN (“BirdLife International and Handbook of the Birds of the World (2016) 2016. *Anser anser*. The IUCN Red List of Threatened Species. Version 2018-1”).

The wild-collected graylag samples derive widely from Eurasia (Figure 1, Supplementary File 1, Table S1) representing both subspecies. As no morphological data were available, we could not discriminate the samples between eastern and western subspecies. However, based on their sampling and the known geographic distribution of the populations, we can be confident that the Iranian and Kazakhstani samples belonged to the eastern subspecies *rubrirostris*. The European domestic goose samples represented 14 different breeds (Supplementary File 1, Table S1) together with individuals unattributed to a recognized breed or which were presumptive hybrids between European and Chinese domestic geese. Some specimens were reported to be Chinese domestic geese. The domestic samples were obtained from local breeders in Denmark, Sweden, and the UK, and those from Turkey were collected directly by the authors.

### DNA extraction and GBS library construction

GBS (Elshire *et al.* 2011) libraries were constructed at the Cornell Biotechnology Resource Center (BRC) following DNA extraction with the DNeasy Blood and Tissue Kit (QIAGEN) with RNase treatment. Each individual DNA sample and an adaptor with a unique barcode were combined in a 96-well plate along with a common adaptor. Samples were treated with the EcoT-22I (ATGCAT) restriction enzyme to create fragmented DNA. Barcoded adapters and common adapters with matching sticky ends were ligated to each sample with T4 DNA ligase. The samples were pooled and purified with a QIAquick PCR Purification Kit (QIAGEN). PCR amplification of the library used primers complementary to barcoded and common adapters with products purified as above, and the samples were 100 bp SE-sequenced with Illumina HiSeq 2000/2500 at the BRC.

### GBS pipeline and SNP calling

Raw sequence reads were run through the Command Line Interface of the Tassel 5 GBS v2 Discovery and Production pipelines (Glaubitz *et al.* 2014). Details about the pipelines and SNP calling are in the Supplementary File 1 (see Figure S1 for quick outline of the workflow). Good quality reads were recorded as tags and aligned to the *A. cygnoid domesticus* GenBank assembly (AnsCyg_PRJNA183603_v1.0 GCF_000971095.1) (Lu *et al.* 2015) using the Burrows-Wheeler Aligner with default settings (Li and Durbin 2009). After running the raw data through the pipelines, 69,865 SNPs were obtained.

The SNPs were subjected to additional filtering using VCFtools (Danecek *et al.* 2011). We removed indels, loci with more than two alleles and invariant loci. However, loci that were within-species invariant but divergent from the reference were retained for phylogenetics, informing about graylag-swan goose divergence. After preliminary analyses loci with observed heterozygosity over 0.75 were removed as potential paralogs. Individuals with more than 20% missing data across loci were removed. The final dataset consisted of 33,527 biallelic SNPs and 133 individuals (58 wild and 75 domestic).

### The estimation of genetic diversity

Genetic diversity and pairwise F_ST_ values were investigated with the hierfstat R package (Goudet 2005). Expected heterozygosity (H_E_) was calculated for each locus and population and averaged across loci. Difference in average H_E_ between graylags and European domestics was tested with a two-sample *t*-test with the Welch correction for non-homogeneity of variance (Welch 1938). For comparing the genetic diversity among wild and domestics, only pure graylag populations (defined as having < 10% admixture with domestic geese) and pure European domestic geese (defined as having < 10% admixture with Chinese domestic geese) were used to avoid hybridization effects on the estimates. The admixture proportions were obtained from STRUCTURE.

The variance components across loci for hierarchical F-statistics for pure graylags and pure European domestics were estimated using locus-by-locus analysis of molecular variance (AMOVA) implemented in Arlequin 3.5.2.1 (Excoffier and Lischer 2010). The significance was tested with 16 000 permutations.

### Population structure analyses

Population clustering and structure was analyzed with STRUCTURE 2.3.4 (Pritchard *et al.* 2000) and Principal Component Analysis (PCA) (Patterson *et al.* 2006). For the whole dataset, STRUCTURE was run with 1000 burn-in steps followed by 10 000 iterations of MCMC for data collection for *K* = 1-10 allowing admixture with five replicates of each run to reach convergence. For the STRUCTURE analyses done separately on graylags and European domestic geese, see Supplementary File 1. An admixture model with correlated allele frequencies among populations (Falush *et al.* 2003) was used in all STRUCTURE analyses and the iterations were automated with StrAuto 1.0 (Chhatre and Emerson 2017). We applied both likelihood of *K* and Evanno’s Δ*K* (Evanno *et al.* 2005) of successive *K* values to determine the optimal number of clusters, using STRUCTURE HARVESTER (Earl and VonHoldt 2012). CLUMPP 1.1.2 (Jakobsson and Rosenberg 2007) was used to align the assignments from different replicates of *K* and distruct 1.1 (Rosenberg 2003) for visualization. A PCA was performed with the prcomp function in R (R Core Team 2017) and the significance of eigenvalues determined based on the Tracy-Widom distribution (Patterson *et al.* 2006; van Heerwaarden *et al.* 2011).

A neighbor-joining tree was constructed for phylogenetic analysis, with pairwise distance between individuals obtained with the R package ape (Paradis *et al.* 2004) based on 40,191 loci. The *A. cygnoid* reference genome and the invariant sites that differed from it were included in the tree construction.

### Tests for admixture and simulations of demographic history

The history of admixture was tested with a 3-Population test *ƒ*_3_(C; A, B) implemented in AdmixTools 4.1 (Patterson *et al.* 2012). This method offers a formal test to explain observed patterns of admixture in a target population without an outgroup. For identification of admixture between Chinese and European domestics, Gray and White Chinese were combined to represent the Chinese, and the Landes breed that had minimum indication of admixture in STRUCTURE was chosen to represent the European domestic source population. In addition, we tested several combinations of graylag geese, European domestic geese, and Chinese domestic geese as source populations to detect possible admixture in populations and breeds that implied admixture in STRUCTURE. See also Supplementary File 1 for further information.

Different models of demographic history were tested with fastsimcoal2 ver 2.6 (Excoffier *et al.* 2013). Fastsimcoal2 uses coalescent simulations to estimate the likelihood of a demographic model and the probabilities obtained from simulations are then used to compute the composite likelihood of the model. The likelihood is maximized with a conditional maximization algorithm (ECM). We excluded all SNPs that had missing data within the whole data set and executed the analyses with a site frequency spectrum (SFS) based on 6,229 SNPs (Supplementary File 1, Figure S2). As there are no estimates of the genetic diversity per base pair for graylags, we estimated the proportions of variable and monomorphic sites in the data as we needed the information about the invariant sites for the fastsimcoal2 analysis. From the BAM file with –depth option in SAMtools 1.7 (Li *et al.* 2009), we estimated 9,801,382 bp covered with GBS tags. We then mimicked the filtering steps done for the biallelic SNPs to reduce the total number of sites in equivalent proportions. We removed the same number of sites that corresponded to the number of SNPs that were removed because they were indels, had more than 2 alleles or had heterozygosity over 0.75. Since some of the SNPs were removed from this analysis due to missing data in some individuals, we removed an equal proportion of sites from the total number of sites as well. The final folded SFS had 1,681,316 sites of which 1,675,087 were monomorphic and 6,229 polymorphic.

**Figure 2:**
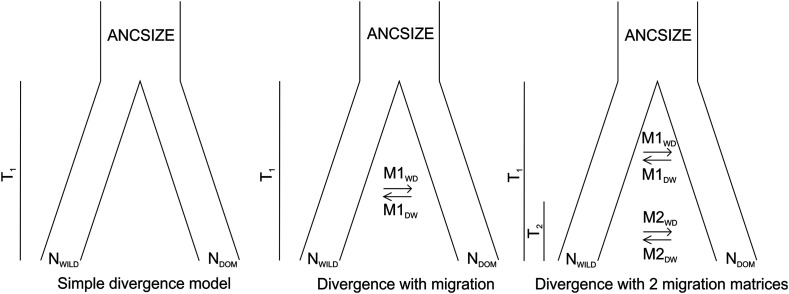
Demographic histories of goose domestication as tested with fastsimcoal2.

To infer the demographic history, we chose a subset of individuals from both wild-collected graylags and domestic geese to represent the genetic variation in both groups. Therefore, 11 graylags with > 90.8% of graylag ancestry and 15 domestic geese with > 91.4% of European domestic goose ancestry were selected for the analysis. The mutation rate for the simulations was 1.38⋅10^−7^ per generation (Pujolar *et al.* 2018). The parameter estimation for each model tested involved 100,000 simulations and 40 conditional maximization (ECM) cycles. The parameters for each model were estimated with 100 independent runs to obtain the global maximum. The models tested were i) simple divergence of two populations with no gene flow, ii) divergence of two populations with continuous gene flow and iii) divergence of two populations with changing gene flow patterns (Figure 2, Figure S3-S4). The best model was selected based on Akaike’s weight of evidence as in Excoffier *et al.* (2013). For parametric bootstrapping 100 SFS were simulated with the parameter estimates obtained from the real SFS, followed by maximum likelihood estimation with 50 independent runs for each bootstrap SFS. The 95% confidence intervals were obtained from the bootstrap data for each estimated parameter.

### Data availability

The Supplementary File 1 that contains extended Materials and Methods, and Results including supplementary figures and tables, and Supplementary File 2 containing commands for the Tassel pipeline and vcftools are stored in figshare along with the VCF file containing the filtered genotypes. The raw sequence reads are available in NCBI’s Sequence Read Archive (SRA) under BioProject PRJNA634849. Supplemental material available at figshare: https://doi.org/10.25387/g3.12594230.

## Results

### Population structure

**Figure 3:**
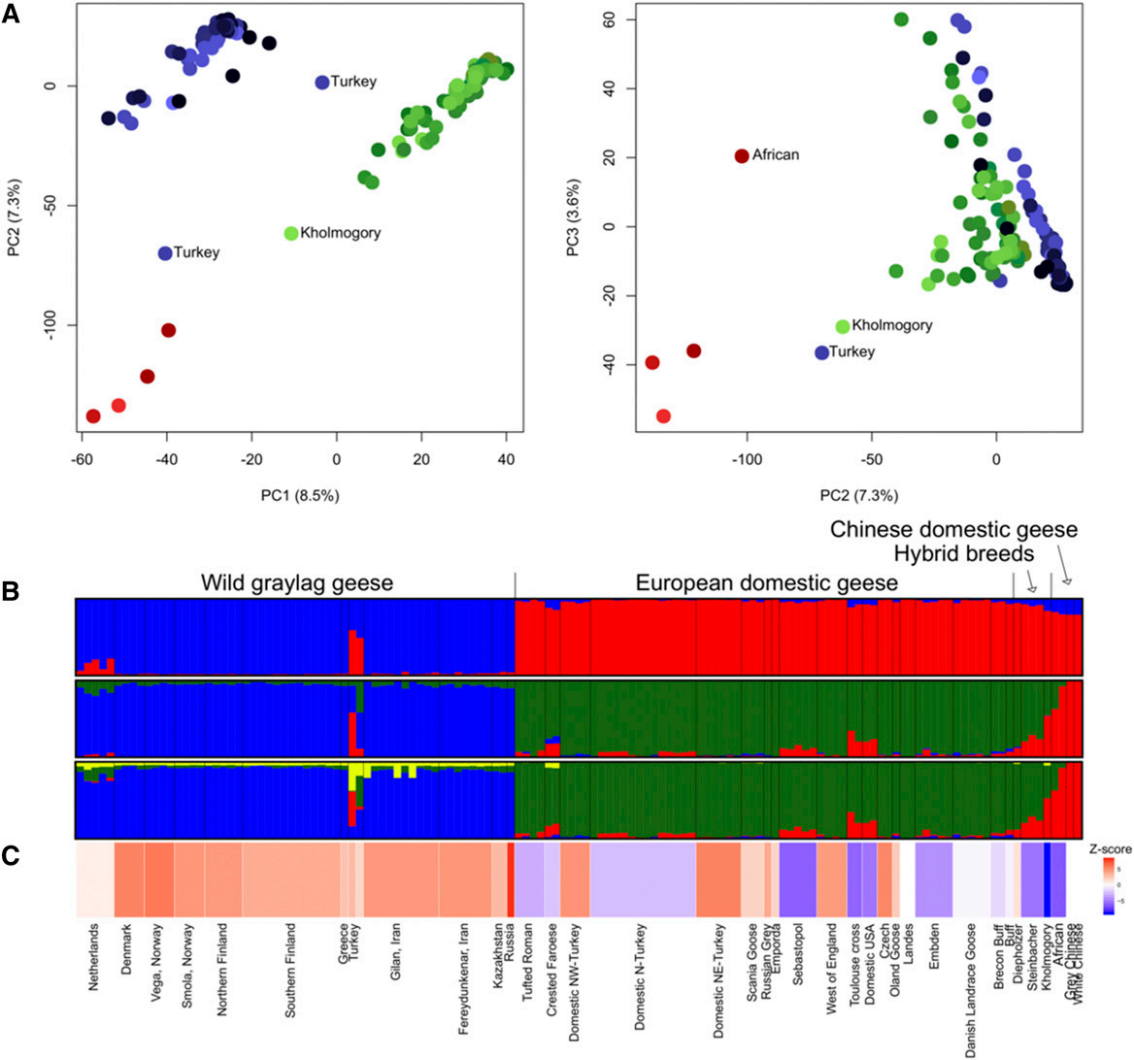
The genetic divergence and hybridization patterns in graylag and domestic geese. Population status and names labeled as in Supplementary File 1, Table S1. The colors in A) and B) are associated to different groups as follows: graylags (blue), European domestics (green) and Chinese domestics (red). A) The first three principal components summarizing the genetic variation in geese (percentage explained by each PC is shown). Different shades refer to different populations. B) STRUCTURE assignment plots for *K* = 2, *K* = 3, and *K* = 4. Each vertical bar represents one individual with *K* number of colors indicating proportion of ancestry from the inferred clusters, and populations/breeds are separated by black vertical line. C) Plot relating to the f_3_ (Supplementary File 1, Table S5) values obtained for each population. Turkey refers to two adjacent bars in the plot since the Turkish graylags were analyzed as two separate individuals. The more negative the f_3_, the more significant is Z-score in favor of admixture. The f_3_ values were not calculated for Landes and the Chinese geese, as they were used as source populations, thus they were given an f_3_ value of 0.

There was clear genetic differentiation between graylags and domestic geese according to STRUCTURE and PCA (Figure 3A-B). STRUCTURE aims to find the optimal number of ancestral populations (*K*) from the given data and the subdivision was clear in our data. At *K* = 2, populations/breeds are clustered based on their status (wild or domestic) and, at *K* = 3, domestic geese are further separated into European and Chinese. At *K* = 4, the fourth cluster is within graylag populations but none of the individuals are unanimously assigned to that cluster. The likelihood was highest for *K* = 3. These results were supported by PCA as the first two PCs out of 14 significant PCs (*P* < 0.05) were enough to separate the three groups (wild, European domestic, Chinese domestic) from each other (Figure 3A). Overall, the graylag populations showed 3.2–23.5% admixture proportions with European domestic geese when *K* = 3 (Table S1). In contrast, not all European domestic geese showed admixture with graylags and the admixture percentages ranged from 0.0 to 8.4%. At *K* = 3 many European domestic goose breeds showed mixed ancestry with Chinese domestic geese (0.0–27.1%).

The neighbor-joining tree repeated the major patterns observed with STRUCTURE and PCA, revealing a star shaped phylogeny and confirming that the domestic and graylag geese largely form different clades (Figure S5). Surprisingly, the Chinese domestic geese were closer to European domestic geese and graylags, than to the swan goose reference genome. In addition, one graylag from Turkey was more closely related to the Chinese domestic geese than other graylags, also indicated by admixture proportions from STRUCTURE. Further, two Crested Faroese individuals and four domestics from the USA (2 unknown and 2 Toulouse crosses) were closer to Chinese than European domestic geese. These six individuals also showed high proportions of admixture with Chinese domestics in the STRUCTURE analysis.

Unequal sample sizes did not have a large effect on the results (Supplementary File 1, Figure S6-S11). Some further population structure was observed within both graylags and domestic geese, when analyzed separately with STRUCTURE and PCA. Geographically, graylags differentiated by subspecies (Supplementary File 1, Figure S12-S13). STRUCTURE indicated little differentiation among European domestic geese, but the PCA revealed separation between the European breeds and the Turkish domestic geese (Supplementary File 1, Figure S14-S15).

### Genetic diversity

An AMOVA was used to partition genetic diversity among graylag *vs.* domestic (group level), and among populations (graylag) and among breeds (domestic), and within population levels (Table 1). The fixation index between graylag and domestic geese was 0.158 and there was also significant differentiation among graylag populations/domestic breeds (Table 1). The average pairwise *F_ST_* between graylag populations and domestic breeds was 0.197, among graylag populations 0.088 and among domestic breeds 0.174 (Supplementary File 1, Table S2).

**Table 1:** Hierarchical analysis of molecular variance (AMOVA) of graylags and their domestic descendants, considering pure populations of graylags (first group) and pure breeds of European domestic geese (second group)

Source of variation	Sum of squares	Variance components	Percentage variation	Fixation indices
Among groups	47565.119	431.4291	15.8	*F_CT_* = 0.158*^a^*
Among populations and breeds within groups	82960.489	302.51404	11.1	*F_SC_* = 0.131*^a^*
Within populations and breeds	345889.821	2003.45893	73.2	*F_ST_* = 0.268*^a^*
Total	476415.429	2737.40207		

a *P* < 0.001.

The genetic diversity measured as average H_E_ was higher in pure graylags (0.146) than in pure European domestic geese (0.096) (Welch’s *t*-test, degrees of freedom (df) = 10.594, *P* = 3.91×10^−5^, see also Supplementary File 1, Figure S16). The average H_E_ ranged from 0.140 (Denmark) to 0.150 (Kazakhstan) in pure graylags and from 0.047 (Landes) to 0.123 (Domestic N-Turkey) in pure European domestics. The difference in average H_E_ remained when non-pure graylag and non-pure European domestics were included in the comparison (0.156 *vs.* 0.107; Welch’s *t*-test, df = 19.28, *P* = 0.000418). The average H_E_ was higher in admixed populations compared to non-admixed populations in both graylag and domestic populations (Supplementary File 1; Table S1, Figure S16).

### Admixture and the time of domestication

STRUCTURE implied considerable mixed ancestry from multiple genetic clusters for Dutch and Turkish graylags, but the *ƒ*_3_ analysis did not confirm admixture for the Dutch population even though multiple source populations of graylag and domestic goose were tested (Table S3). However, the Turkish population is more complicated as they obtained negative *ƒ*_3_ when analyzed together with multiple combinations of source populations indicating admixture with Chinese domestic goose but not with European domestic goose. This signal appeared consistently when several graylag and European domestic goose populations were used as source populations with Chinese domestic geese. However, as the Turkish graylags appeared genetically very dissimilar, we analyzed them separately which resulted in neither of them obtaining negative *ƒ*_3_ (Table S3). The two Turkish graylag samples came from the same area as our NW-Turkish domestic population, which among Turkish domestic geese showed highest admixture with graylags (2.2%), but admixture was not confirmed with the *ƒ*_3_ test (Table S4). We did not obtain negative Z-scores to any of the other graylag populations either (Table S5-S6).

The *ƒ*_3_ analysis confirmed admixture of domestic geese in line with the STRUCTURE results. Most notably, the African breed is a hybrid between European and Chinese domestic geese (Z-score -6.399), unexpected as this breed has been assumed to have originated solely from swan goose. The European-Chinese hybrid status of the Kholmogory and Steinbacher breeds was also confirmed (Z-scores of -8.933 and -5.349, respectively). The Kholmogory breed also fell halfway between European and domestic geese both in STRUCTURE and PCA, whereas the Steinbacher was genetically closer to European domestic geese in the PCA. However, the Diepholzer breed, which reportedly is also a hybrid, was not confirmed as such in our analysis. Other domestic breeds/groups with admixture status in STRUCTURE were also confirmed to have a European-Chinese admixture when a Z-score threshold of -3 (roughly corresponding to *P* < 0.01) was used: Sebastopol, Toulouse cross, Domestic NY, Embden, Tufted Roman (Figure 3C, Supplementary File 1, Table S5). These breeds also gave a similar signal when other combinations of European domestic goose breeds and Chinese domestic geese were used as source populations (Table S7). The Crested Faroese breed gave indication of admixture based on STRUCTURE analysis and the *ƒ*_3_ test supported this (Z-score of -2.228, *P* < 0.05). Surprisingly, the Northern Turkish domestic population was not admixed with Chinese domestic geese in STRUCTURE but *ƒ*_3_ analysis gave a contrasting signal (Z-score -2.459, *P* < 0.05).

The demographic model that best fit our data suggested divergence of graylag and domestic geese with a recent migration rate change (Table 2, Supplementary File 1, Table S8). The model suggested divergence around 5319 generations ago (95% confidence intervals (CI): 2014-6503) with asymmetric but close to equal migration rates from graylags to domestic geese following divergence. About 159 (88-476) generations ago, there was a change in the gene flow patterns, suggesting higher gene flow (m) from graylag geese to domestic geese toward modern times. However, translated to actual number of migrants (N_e_m), the numbers suggest that the gene flow has been higher from domestic geese to graylag geese across domestication history, (0.41 graylag geese *vs.* 1.34 domestic geese migrating per generation following the domestication event, and 1.65 graylag geese *vs.* 1.67 domestic geese per generation migrating after the gene flow pattern changed). Given an estimated generation time for these geese of about 3 years, the numbers suggest divergence about 14 000 BCE and gene flow shift about 480 years ago.

**Table 2:** Maximum likelihood estimates (MLE) for the parameters of the preferred demographic model for goose domestication history (see text) with their 95% confidence intervals (CI)

Model	Parameter	MLE	95% CI
Divergence with changing gene flow patterns	ANCSIZE	1112	378.95 - 7990.65
	T_1_	5319	2014.45 - 6503.75
	M1_WD_	4.25x10^−4^	1.21x10^−7^ - 6.28x10^−4^
	M1_DW_	5.35x10^−4^	2.88x10^−4^ - 6.45x10^−4^
	T_2_	159	88.9 - 476.25
	M2_WD_	1.72x10^−3^	1.30x10^−3^ - 2.23x10^−3^
	M2_DW_	6.69x10^−4^	4.17x10^−4^ - 8.00x10^−4^
	N_WILD_	2504	2352.4 - 2680.25
	N_DOM_	959	833.95 - 1040.55

ANCSIZE, effective population size of ancestral population; T_1_, time of divergence in generations; N_DOM_, effective population size for domestic geese; N_WILD_, effective population size for graylags; T_2_, estimate of time in generations when the migration matrix switched; M1_WD_ migration rate from wild to domestic following T_1_; M1_DW_ migration rate from domestic to wild following T_1_; M2_WD_ migration rate from wild to domestic following T_2_; M2_DW_ migration rate from domestic to wild following T_2_.

## Discussion

We studied the dynamics of domestication and hybridization in gray (*Anser*) geese using genome-wide SNP data. The results demonstrated genetic divergence between Eurasian wild graylag and European domestic geese with long-term genetic exchange between them. We also inferred temporal changes in the direction of gene flow. The degree of hybridization between graylag and domestic geese also varied geographically. Surprisingly, several domestic goose breeds also showed a substantial genetic contribution of Chinese domestic geese. We also provide insights about the origin and the timing of goose domestication.

### Genetic diversity and differentiation of graylag and European domestic geese

Domestic species often show reduced genetic diversity compared to their wild ancestor, attributable to genetic drift during population bottlenecks of initial domestication, combined with subsequent artificial selection associated with breed formation (Moyers *et al.* 2018). Domestic geese appear to follow the same trend. We found European domestic geese to have lower H_E_ than wild graylags. In general, graylag populations were much more uniform in their level of genetic diversity whereas domestic populations showed more variance, which is likely to reflect the human influence on breed formation.

European domestic geese are genetically distinct from their wild progenitor but no more so than for other domestic birds. The average pairwise *F_ST_* values between graylag populations and domestic goose breeds were lower than between red junglefowl and domestic chicken populations (Kanginakudru *et al.* 2008), and domestic geese are less distinctive than domestic pigeons (Stringham *et al.* 2012). Among domestic geese, the Turkish are particularly interesting. From mtDNA, the Turkish domestic geese stand out as the most genetically variable group (Heikkinen *et al.* 2015), and although this is less evident from GBS, among the pure European domestic geese the Northern Turkish showed the highest average H_E_. The *ƒ*_3_ analysis indicates a history of admixture with Chinese domestics for this population, which may explain its high genetic diversity.

We found a genetic separation between European and Near Eastern populations of graylags that aligned with the western and eastern subspecies (*A. a. anser* and *A. a. rubrirostris*) (Scott and Rose 1996), a distinction which could not be made based on mtDNA (Heikkinen *et al.* 2015). Hybridization between the western and eastern subspecies is suggested from admixture in Dutch and Danish graylags in STRUCTURE as there is a genetic component that is more prevalent in the eastern populations. There is historical evidence for the introduction of *rubrirostris* to Belgium in 1954 and to Netherlands in 1960s (Rooth 1971; Kuijken and Devos 1996); thus, *rubrirostris* genes may have originated from the recently introduced gene pool spreading to Denmark.

### When and where were geese domesticated?

Traditional views on goose domestication claim it first occurred in the eastern Mediterranean (possibly Egypt) around the 3^rd^ Millennium BCE (Zeuner 1963; Albarella 2005). Domestication of chicken and perhaps pigeon took place earlier, but domestication of duck later, at least in Europe (Larson and Fuller 2014). Demographic modeling suggests that the wild graylag and related domestic lineages split approximately 5,300 generations ago placing domestication origins at 14 000 BCE assuming a 3-year generation time (Cramp and Simmons 1977). This estimated genetic divergence time is, admittedly, considerably earlier than any evidence for animal domestication except dog. It is important to note that the estimated divergence times have large confidence intervals and merely indicates the split between the ancestors of contemporary wild and domestic lineages. It is most likely that our demographic modeling reflects the early divergence of different lineages of graylags, only one of which contributed to later domestication. The subsequent reduction or even disappearance of that wild lineage means that, despite wide geographical sampling, the possible modern wild population(s) of the graylag progenitor to domestic geese was not sampled in this study. It is also worth remembering that using *A. cygnoid* reference genome may have caused a mapping bias of *A. anser* alleles failing to map on the reference genome due to sequence divergence. This would have affected the subsequent SNP calling by reducing the number of rare, derived *A. anser* alleles, which in turn could cause our divergence time estimate to be an underestimate. Another thing to bear in mind is the uncertainty about the mutation rate. The estimate we used by Pujolar *et al.* (2018) was estimated for pink-footed goose which is a closely related to graylag goose and was supported by Ottenburghs *et al.* (2016b) who obtained a similar substitution rate for geese. However, both estimates are about two orders of magnitude higher than that estimated for collared flycatcher using pedigree data (Smeds *et al.* 2016). It is possible that this is a taxon-related difference but in case the substitution rate for graylag goose is actually closer to that of collared flycatcher, the mutation rate we used here would be too high and our estimate of the domestication time would have to be pushed even further back. Therefore, the estimated divergence time should be considered as a guideline for future studies and not as an absolute truth. Future studies would benefit from whole genome sequencing of graylag goose in resolving the questions about both mapping bias and the substitution rate.

Given that genetic diversity would be expected to be highest in the ‘domestication center’ and reduce with increasing distance from there, the high mtDNA diversity of Turkish domestic geese means the eastern Mediterranean cannot be ruled out as a candidate for the origin of goose domestication. However, as we have shown, hybridization between wild and domestic geese can also generate high genetic diversity both within and outside the original domestication location. More thorough sampling of the graylag population around the Black Sea would be beneficial in resolving the role of eastern Mediterranean region in the domestication history of goose as this population was not well represented in our study. Additionally, the progenitor of domestic geese could be sought by ancient DNA approaches.

### The role of intra- and interspecific hybridization in goose domestication history

#### Evidence of current hybridization:

Domestic animals and their wild relatives are often observed to interbreed, and this is also true for geese. Both field observations and mtDNA results (Kuijken and Devos 1996; Heikkinen *et al.* 2015) suggested some current hybridization between domestic and graylag geese. Genome-wide analysis covering multiple graylag populations and domestic breeds revealed a considerable impact of hybridization on genetic diversity of both wild and domestic geese.

Hybridization is particularly prevalent in certain geographical regions. Dutch and especially Turkish wild graylag samples had more shared genetic affiliation with domestics than Scandinavian and Finnish graylag populations (Figure 3B). Some regions may offer more hybridization opportunities, *e.g.*, climate may allow graylags to be sedentary year-round and be favorable for keeping domestic geese. The Netherlands, for instance, lies on the Atlantic flyway offering breeding, staging, and wintering areas for graylags (Madsen *et al.* 1999; Andersson *et al.* 2001). Since pair-bonding of geese generally occurs on wintering grounds (Rohwer and Anderson 1988), hubs for migrating geese such as the Netherlands may permit populationmingling. Nevertheless, the *ƒ*_3_ test did not support a simple history of admixture for the Netherlands. Patterson *et al.* (2012) have stated that population-specific drift may mask the signal of admixture in such analyses, leading to a non-negative *ƒ*_3_. The *ƒ*_3_ model is relatively simple, with only two sources, and may not catch the signal of admixture in the Dutch graylag population because of the previous contribution of *rubrirostris*, which was not included in the model.

Based on ringing data most graylag populations in Scandinavia follow the Atlantic flyway - some of the geese wintering in the Netherlands and others in southwest Spain. However, Finnish graylags favor the Central European flyway and winter in North Africa, with a minority of Finnish graylags using the Atlantic Flyway (Madsen *et al.* 1999; Andersson *et al.* 2001). The Finnish populations of graylag showed the lowest admixture proportions with domestic geese (S-Finland 3.2% domestic goose, N-Finland 3.3% domestic geese) among graylag populations. Rearing geese is not a popular practice in Finland, and they constitute less than 5% of poultry kept in Finland (“Official Statistics of Finland (OSF): Number of livestock [e-publication]. Helsinki: Natural Resources Institute Finland [referred: 22.7.2020]. Access method: http://www.stat.fi/til/klm/index_en.html” 2020). The Norwegian populations showed only slightly higher admixture proportions with domestic geese, although the domestic mtDNA haplotype ANS19 was detected from a wild graylag collected in Finnmark, Norway (Pellegrino *et al.* 2015). This haplotype is a partial sequence of the D5 haplotype identified by Heikkinen *et al.* (2015), and identical to that found in White Roman domestic geese (Wang *et al.* 2010).

Inferring the hybridization patterns in the Turkish graylags is more complicated, as Turkish graylags indicate hybridization with both Chinese and European domestics. Both graylags sampled in Turkey showed considerable admixture with domestic geese. One of them appeared genetically as a hybrid of European and Chinese domestic goose with only a small proportion of graylag ancestry, whereas the other one was a more equal mix of European domestic goose and graylag supplemented by a considerable Chinese domestic goose ancestry. However, what appears as a hybridization between European and Chinese domestic geese may also be related to ancestral variation, and result from close relatedness of the Turkish graylags to the graylag population that was domesticated, reinforced by a gene flow from the Chinese domestic goose. There is some indication of hybridization between graylags and domestic geese within that area as the domestic geese sampled from the same area showed some admixture with graylags, but this was not confirmed with *ƒ*_3_ analysis. These results may reflect a local practice of keeping captive graylags within a flock of domestic geese as several sources state that it has been a common practice to collect wild eggs and goslings in many places across Eurasia (Gray 1871; Honka *et al.* 2018). Another possibility is that the Turkish graylags have hybridized with some unsampled distinct graylag population and simply appear genetically like domestic geese due to lack of representation of the unsampled wild population. The graylag population breeding and wintering in the Black Sea region is not well monitored (Fox *et al.* 2010).

#### Long-term hybridization:

Domestication can be seen as an analogy of speciation where an animal population transforms to an ecotype that is adapted to the human niche (Larson and Fuller 2014) and at later stages of domestication is perpetuated with reproductive isolation in the form of selection managed by humans (Zeder 2012). However, this reproductive isolation may not be complete (Frantz *et al.* 2015). While the genetic divergence of the graylag and its domestic descendant is evident, our results suggest extensive long-term genetic exchange between them. In addition, the demographic modeling suggests that the gene flow patterns have changed over time.

Initially, gene flow was greater from domestic geese to graylag geese. It is unlikely that the early stages of goose domestication were rigorously managed, allowing matings outside the domestic gene pool. It is in the farmers’ interest to keep the domestic geese and wild geese reproductively isolated to keep control over the traits that are being selected, but artificial selection of traits would have become possible only after the domestic gene pool had been established. After that, it may occasionally be beneficial to restock the flock to maintain enough genetic diversity. Several sources have suggested that it has been a common practice to collect goose eggs from the wild and raise them in captivity. The natural tendency for imprinting in geese facilitates this practice. Goose-keeping became well-established in the Medieval period (Albarella 2005) and the rise in number of domestic geese may have allowed an increase in domestic goose escapees resulting in increased gene flow (N_e_m) from domestic geese back to graylags toward modern times.

Furthermore, not only have domestic geese admixed with wild graylags but also European and Chinese domestic geese have hybridized. Hybridization with ancestral species or closely related species is frequent in domestic species, *e.g.*, the genetic composition of chicken derives from multiple different species of *Gallus* (Eriksson *et al.* 2008). Similarly, the genetic composition of domestic geese seems to derive from two closely related species. This hybridization with Chinese domestic geese may have introduced some traits not present in graylags to European domestic geese and vice versa.

## Conclusion

This study is the first attempt to answer questions related to goose domestication history using population genetic approach with genome-wide data. We have shown that hybridization has played and continues to play a significant role in shaping the wild and domestic graylag populations. Admittedly, the demographic models we used here were quite simple and they are unlikely to capture every nuance of the population history, but they offer a starting point for future studies which may include more elaborate analyses of demographic history, for example changes in effective population size associated with population bottlenecks during domestication. Selection scans could be used to identify introgressed alleles that have been under selection during domestication. The use of whole genome sequencing would be advantageous in aforementioned analyses and would also enable assessment of runs of homozygosity (ROH) in goose genome.
